# Preparation and characterizations of chitosan–octanoate nanoparticles for efficient delivery of curcumin into prostate cancer cells

**DOI:** 10.1007/s13205-024-04157-6

**Published:** 2024-11-28

**Authors:** Ahmad Bani-Jaber, Safaa Taha, Rana Abu-Dahab, Samaa Abdullah, Dina El-Sabawi, Alaa A. Al-Masud, Alhassan H. Aodah, Abeer A. Altamimi

**Affiliations:** 1 Department of Pharmaceutics and Pharmaceutical Technology, School of Pharmacy, The University of Jordan, Amman, Jordan; 2 Department of Biopharmaceutics and Clinical Pharmacy, School of Pharmacy, The University of Jordan, Amman, Jordan; 3 Natural and Health Sciences Research Centre, Princess Nourah Bint Abdulrahman University, P.O. Box 84428, 11671 Riyadh, Saudi Arabia; 4 Tissue Banking Section, Research Department, Natural and Health Sciences Research Centre, Princess Nourah Bint Abdulrahman University, P.O. Box 84428, 11671 Riyadh, Saudi Arabia; 5 Advanced Diagnostics and Therapeutics Institute, Health Sector, King Abdulaziz City for Science and Technology (KACST), 11442 Riyadh, Saudi Arabia; 6 Present Address: Samaa Abdullah, College of Pharmacy, Amman Arab University, Amman, 11953 Jordan

**Keywords:** Chitosan, Octanoic acid, Salification, Curcumin, Nanoparticles, Prostate cancer

## Abstract

The goal of the research was to develop a hydrophobic octanoate salt of chitosan (CS–OA) and use the salt as a nanoparticle platform for the delivery of curcumin (CUR) into prostate cancer cells. The nanoprecipitation technique was used to prepare the nanoparticles, which were measured for particle size and encapsulation efficacy relative to CUR–CS nanoparticles. The cytotoxicity of CUR–OA–CS nanoparticles was evaluated in prostate cancerous cells (PC3 and DU145) in comparison with the corresponding blank nanoparticles and hydroalcoholic CUR solution. PXRD, SEM, and TEM were also used to examine the CUR–CS–OA nanoparticles. The average diameters of the CUR–CS–OA and CUR–CS nanoparticles were 268.90 ± 3.77 nm and 221.90 ± 2.79 nm, respectively, with encapsulation efficiencies of 61.37 ± 1.70% and 60.20 ± 3.17%. PXRD and SEM suggested CUR amorphization in the CS–OA nanoparticles. The void nanoparticles exhibited concentration-dependent antiproliferative action, which was attributed to the cellular uptake of CS. CUR loading into these nanoparticles increased their cytotoxicity even more. The potential of CS–OA nanoparticles as a special delivery system for additional cytotoxic drugs into different malignant cells can be further explored.

## Introduction

Curcumin (CUR) is a polyphenol derived from the plant *Curcuma longa*, (turmeric). It has been extensively studied for its potential value in the prevention and treatment of cancer along with its antimicrobial and antioxidant effects (Aggarwal et al. 2003; Zhang et al. 2024b). CUR is safe even at high doses (up to 12 g/day) in humans, but it exhibits poor oral bioavailability due to its poor absorption, rapid metabolism, and pre-systemic elimination. However, CUR nanoparticles, prepared using natural polymers, such as chitosan (CS), showed a promising improvement in their cell membrane permeability and bioavailability. This broadens CUR use as a natural anticancer agent with lesser toxicity when compared to the traditional anticancer drugs in the market (Popat et al. 2014).

Chitosan (CS) is the only pseudo-natural cationic polymer; this unique character reflects the wide variety of applications of CS in medical and pharmaceutical applications (Rinaudo 2006). CS is a natural, nontoxic, and renewable compatible biomaterial. It has exceptional biocompatibility according to its low immunogenicity (Abdullah et al. 2022a; Ali and Ahmed 2018; Bani-Jaber and Abdullah 2020; Md et al. 2021). Its primary amino functional groups give CS distinctive biological properties like muco-adhesion, controlled drug delivery, transfection, in situ gelation, permeation enhancement, and colon targeting (Abdullah et al. 2022a; Bravo-Osuna et al. 2007).

Octanoic acid (OA), also known as caprylic acid, is a saturated medium-chain fatty acid with an 8-carbon backbone and is practically insoluble in water. It is found naturally in the milk of various mammals and is a minor component of coconut oil and palm kernel oil (Scomoroscenco et al. 2023). Moreover, the reported electro-sprayed stearic acid-coated ethylcellulose microparticles for improved sustained release of anticancer drug protocols pioneered an entirely novel approach to create sustained drug delivery hybrids through a combination of insoluble cellulose gels and lipids using modified coaxial electro-spraying (Ji et al. 2023; Dhayalan et al. 2024; Kadian and Rao 2024). Superior biocompatibility, ease of manufacture, scalability, non-toxicity, and targeted distribution are just a few of the appealing benefits of using the hydrophobic octanoate salt of chitosan (CS–OA) for nanoparticles as drug delivery vehicles. Due to their current limitations in drug delivery, more studies are required to fully exploit the potential of lipid-based nanoparticles for potential clinical and therapeutic uses (Dhayalan et al. 2024).

Cancer is a significant public health problem worldwide; despite the rapid advances in diagnosis and treatments, the survival rate from cancer has not improved substantially over the past years (Jemal et al. 2010). Prostate cancer is rated the second most common cancer and the sixth leading cause of cancer deaths among men globally. The signaling cascade of autocrine/paracrine growth factors is essential in promoting prostate cancer cell growth, survival, migration, and progression. At early stages, prostate cancer is hormonal-dependent so it could be controlled with hormone ablation therapy that suppresses the rate of prostate cancer growth. In advanced stages, cancer overcomes its hormone dependence, becomes castration-resistant prostate cancer, and metastasizes to the lung, liver, and bone (Bubendorf et al. 2000).

Clinically, it is approved that drugs can be delivered by binding with drug carriers, such as polymers, in the nanoparticle delivery system of the cytotoxic agent (Abdullah et al. 2022a; Md et al. 2022). The small diameter of the carriers (less than 150 nm) makes them capable of extravagating and localizing to areas of increased vascular permeability. This significantly increases the drug delivered to solid tumors compared to the free drug (Allen 2002).

The salt formation between CS and OA can impart hydrophobicity to the hydrophilic polymers and consequently may improve cellular uptake and nanoparticle system stability (Puro et al. 2006). Meanwhile, new excipients are of high need for drug delivery, the modification of CS to CS–OA salts is a merit (Zhang et al. 2024a). Thus, the main goal of this study was to study ion-pairing between CS and OA and to prepare chitosan–octanoic acid (CS–OA) nanoparticles loaded with CUR as a natural cytotoxic agent. Based on the FT-IR, PXRD, and TEM images, the OA influence was based on stabilizing the interactions between the CUR poorly water-soluble ingredient with the cationic chitosan chains to develop neat and spherical particles. Cytotoxicity of the nanoparticles against dermal fibroblast (HDFa) and prostate cancerous cells (PC3 and DU145) was assessed, in comparison with CS nanoparticles and free CUR solution.

## Results and discussion

### Optimum ratio (OA:CS) of salt formation

**Fig. 1:**
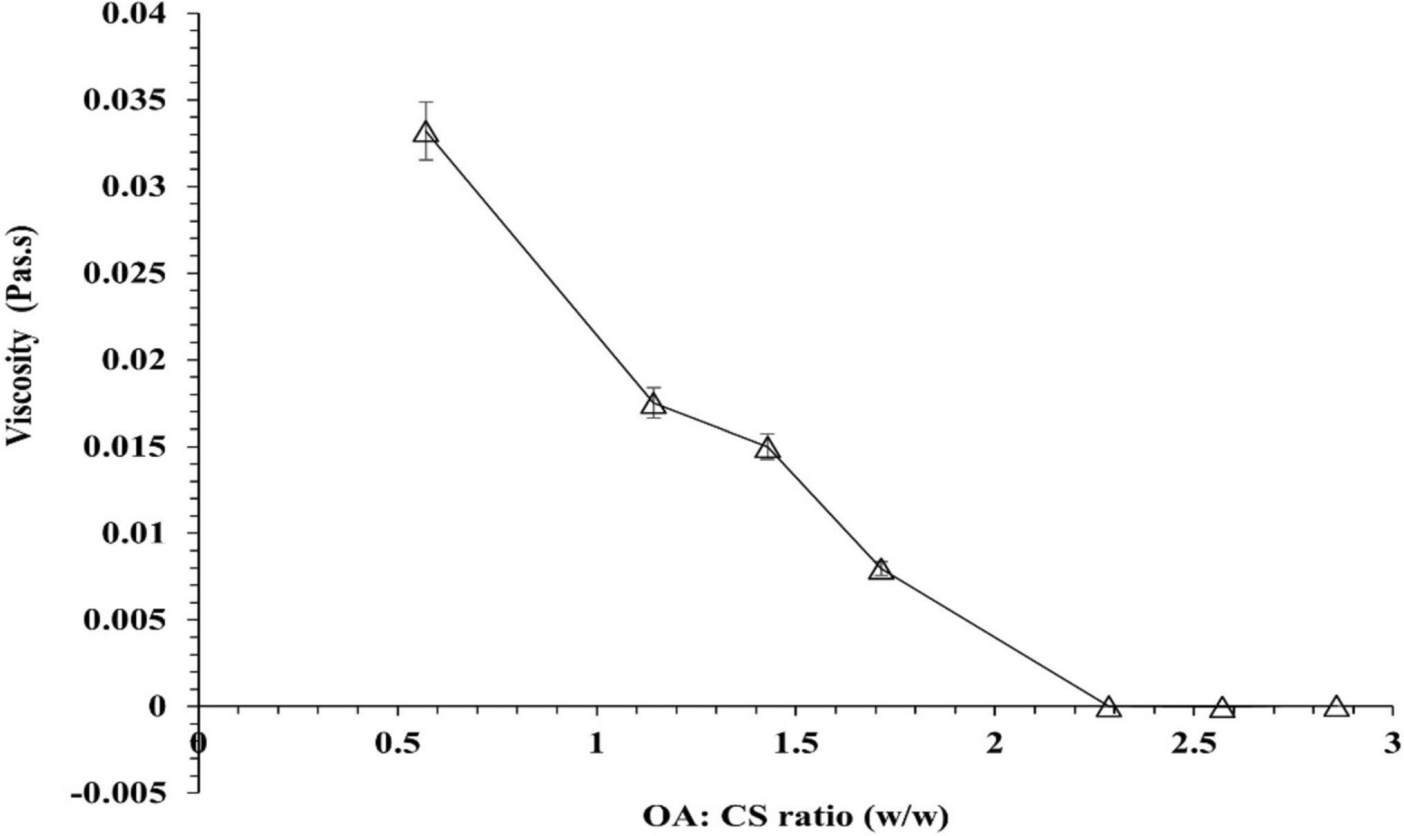
The viscosity of supernatants of CS citrate/K-octanoate mixtures as a function of OA:CS ratio decreases to reach the optimum ratio of 2.30 OA:CS ratio (w/w)

The viscosity of supernatants obtained from OA–CS mixtures as a function of OA:CS weight ratio was given in Fig. 1. Viscosity progressively decreased in an almost linear fashion as the ratio increased from 0.57 to 2.29, beyond which viscosity did not significantly change (P-values were less than 0.05). Accordingly, for every 100 mg CS, nearly 250 mg of OA is needed for the highest yield of CS–OA (Bani-Jaber and Abdullah 2020).

### Characterization of OA–CS salts

**Fig. 2:**
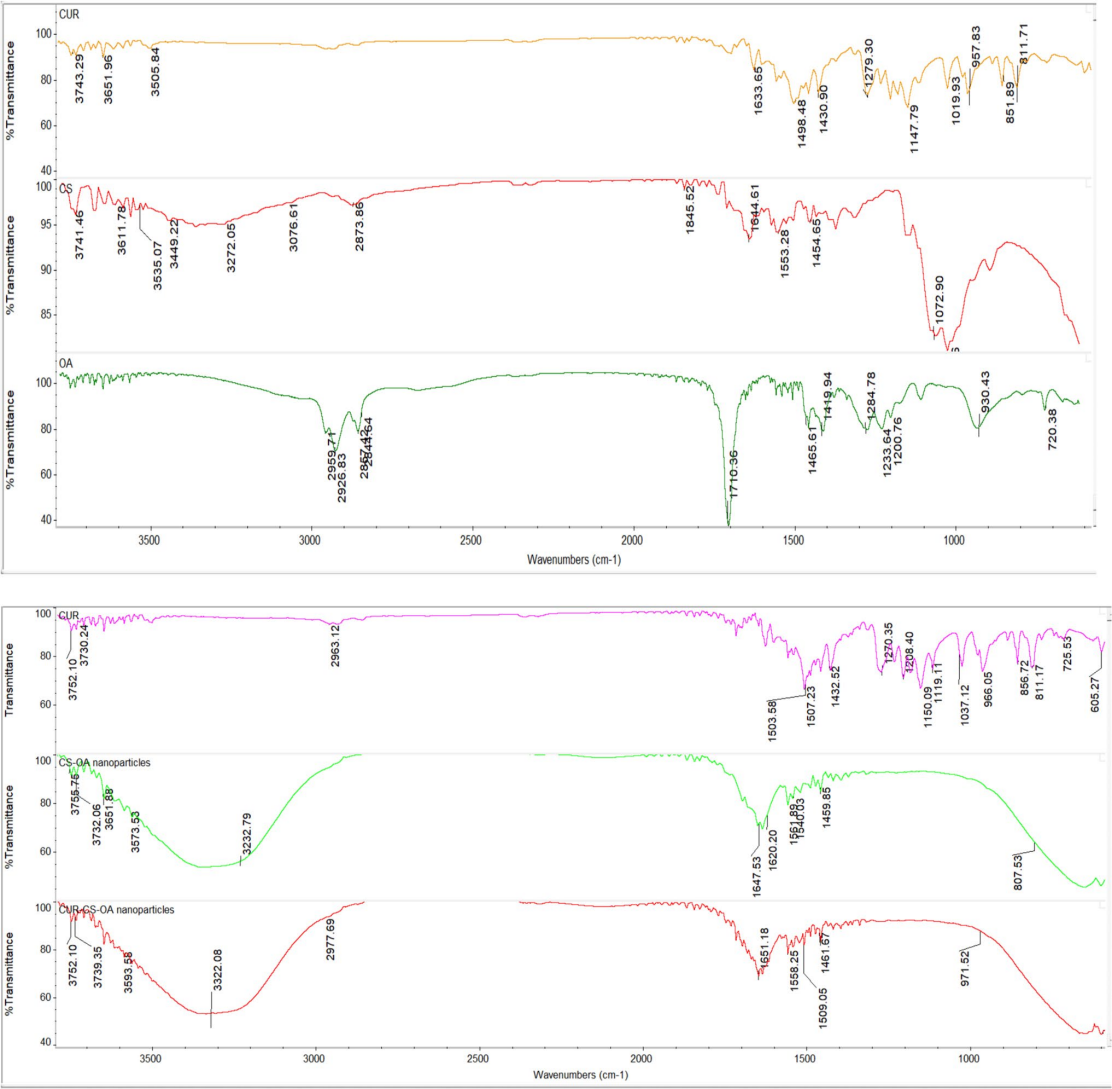
FT-IR spectra of CUR, CS, OA, CS–OA nanoparticles, and CUR–CS–OA. Nanoparticles confirming the OA and CS coating of CUR and hydrogen bond interaction

CS–OA salt prepared using CS citrate by the coprecipitation method was characterized using FT-IR spectroscopy. Its spectrum was compared with those of the CS, OA and CS–OA physical mixture as reported in Fig. 2. The spectrum of CS shows a broad band between 3000 and 3700 cm^−1^ due to O–H and N–H stretching, as well as intramolecular hydrogen bonding. The band at 2864 cm^−1^ was due to C–H stretching. The presence of residual N-acetyl groups was confirmed by the band around 1645 cm^−1^ (C=O stretching of amide I). The CH_2_ bending and CH_3_ symmetrical deformations were confirmed by the presence of bands at 1422 and 1377 cm^−1^, respectively. The absorption band at 1153 cm^−1^ was attributed to asymmetric stretching of the C–O–C bridge. The band at 1025 cm^−1^ corresponded to C–O stretching (Rinaudo 2006). Regarding the spectrum of OA, the carboxylic acid O–H stretching appeared as broadband in the region 3300–2500 cm^−1^, centered at about 2926 cm^−1^. The carbonyl stretching C=O of a carboxylic group appeared as an intense band of 1707 cm^−1^. The C–O stretching and O–H bending appeared in the regions 1320–1210 cm^−1^ and 1440–1395 cm^−1^, respectively. The physical mixture showed the bands for the single components (Puro et al. 2006).

The spectrum of CS–OA coprecipitate, obtained by mixing CS citrate solution and K-octanoate solution, showed a strong peak of 1700 cm^−1^, attributed to the carbonyl group of the carboxylic group in OA, relative to 1707 cm^−1^ shown for OA–CS physical mixture and (Fig. 2). Besides this shift, the peak for the coprecipitate was stronger than that for the PM, which could be a result of the coprecipitation of CS, OA, and citric acid with three carboxylic groups (Bravo-Osuna et al. 2007).

### Nanoparticles preparation

**Fig. 3:**
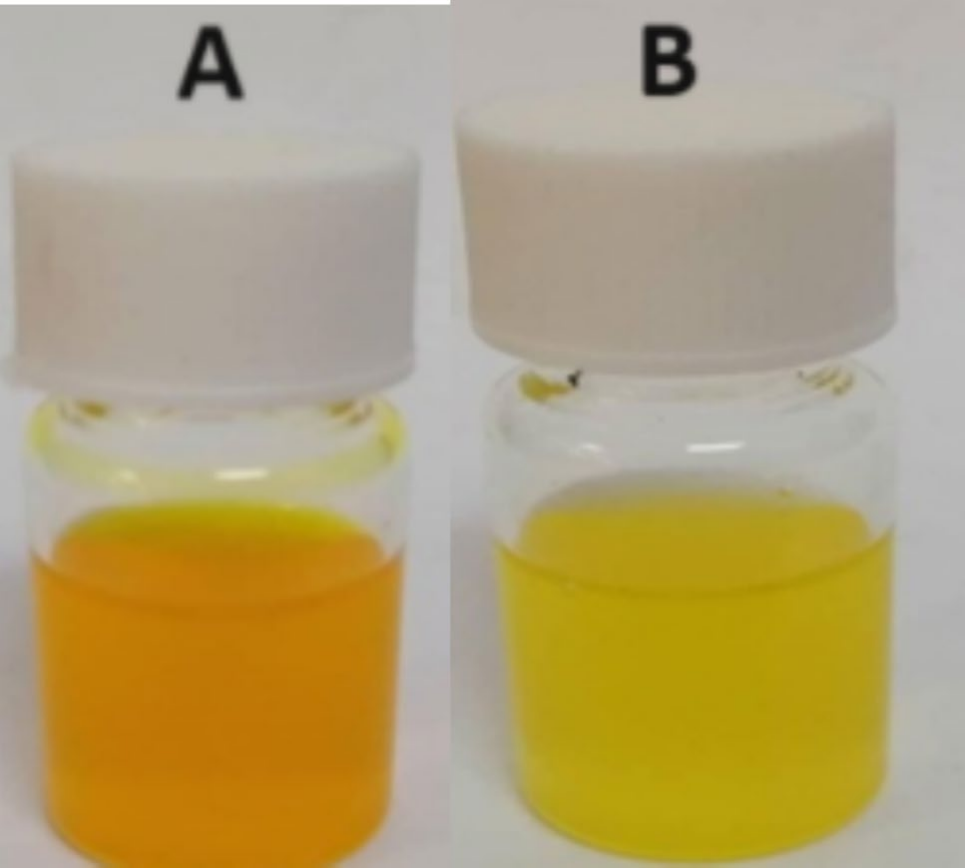
CUR nanoparticles of different colors are prepared as TPP cross-linked CS nanoparticles (**A**) and CS–OA nanoparticles (**B**)

CUR nanoparticles prepared as CS–TPP and CS–octanoate (CS–OA nanoparticles), looked visually translucent homogeneous dispersions and free of aggregate as given in Fig. 3A, B, respectively. However, the orange color is for CS–TPP nanodispersion and yellow for CS–OA nanodispersion. This could be attributed to different counter ions reacted with CS. TPP as a tri-sodium alkaline substance likely rendered the pH of the nanoparticle matrix environment alkaline. The opposite effect is expected for the inclusion of OA in the nanoparticle i.e., acidic effect (Kurczewska 2023; Ali and Ahmed 2018; Popat et al. 2014).

CUR color was different as the pH of the dissolving medium varies based on the following mechanism (Nguyen et al. 2015). CUR structure is a bi-phenol and 1,3-diketone. CUR has an extended conjugation with two aromatic phenolic rings. This tautomerism, extended conjugation and aromatic phenolic rings combined could make CUR yellow. Phenols like in CUR, are chemically acidic but could easily drop their acidic proton in an alkaline environment. The loss of proton at any of the phenolic sites converts the phenolate ion from a benzenoid structure into a quinonoid one. When it switches into quinonoid, the extended conjugation and the tautomerism are altered. Consequently, a bathochromic shift arises in the optical properties, as the quinonoid form appears with a longer wavelength than its benzenoid form as red instead of yellow (Priyadarsini 2014).

### Nanoparticle characterizations

#### Size and zeta potential

**Fig. 4:**
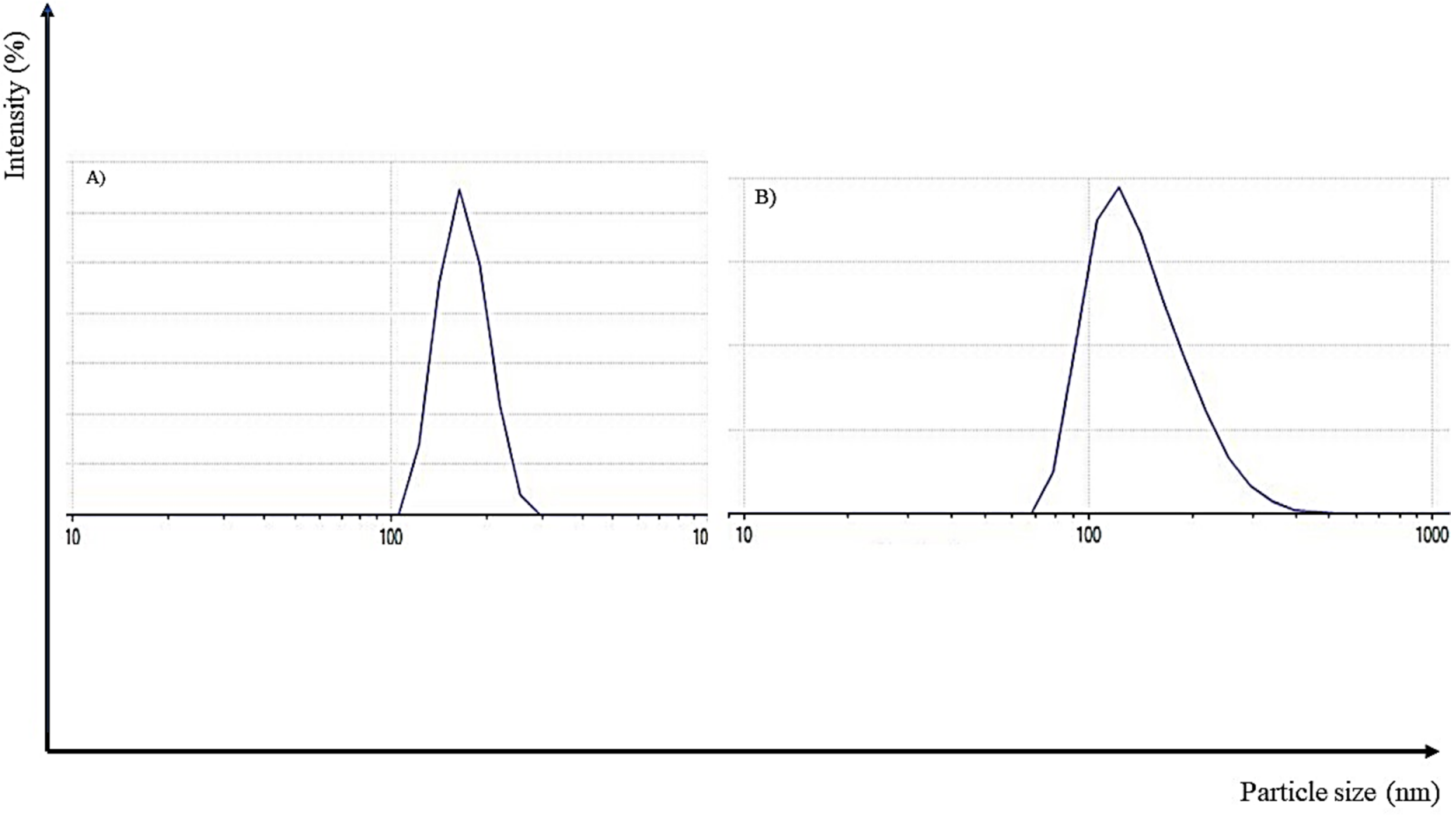
DLS particle size analysis of CUR–CS nanoparticles (**A**) and CUR–CS–OA nanoparticles (**B**). The CUR–CS–OA nanoparticles' size distribution is lower than the CUR–CS nanoparticles

Size analysis results of the prepared nanoparticles by dynamic light scattering are reported in Fig. 4 and Table 1. CS nanoparticles had a mean particle size of 110 nm in the range of 100–200 nm. Relatively, CS–OA nanoparticles had a significantly smaller mean size of 104 nm in the range of 80–300 nm. The size of nanoparticles is a determinant function in both their cellular uptake and biodistribution. Ideally, nanoparticles should avoid rapid clearance from the blood via renal, hepatic and Reticuloendothelial system (RES). On the other hand, too small a size of less than 10 nm can lead to rapid renal clearance (Owens and Peppas 2006).

**Table 1:** Mean particle size (nm), polydispersity index (PDI) and zeta potential (mV) of the nanoparticle

Nanoparticle type	Mean size (nm)	PDI	Zeta potential (mV)
CS nanoparticles	108.90 ± 3.77	0.28 ± 0.01	1.44 ± 0.428
CS–OA nanoparticles	101.90 ± 2.79	0.38 ± 0.02	6.16 ± 0.215

Each value is the mean of *n* = 3 ± Standard Deviations (SD)

Poly-dispersity index (PDI) values of both types of nanoparticles were less than 0.4 indicating monodispersity. On the other hand, zeta potential values of both types of nanoparticles were + 1.44 mV and + 6.16 mV for CS (CS–TPP) and CS–OA nanoparticles, respectively. These small positive zeta potential values could be attributed to the neutralization of CS charge upon crosslinking with TPP or salt formation with octanoate. CS nanoparticles of low zeta potential were previously stabilized by additives that can prevent nanoparticle aggregation (Rampino et al. 2013). For example, polymeric additives could be adsorbed on the surface of nanoparticles in a multimolecular pattern to impact steric stabilization against flocculation and aggregation. Accordingly, PVA and BSA, which were used as stabilizers for CS and CS–OA nanoparticles, respectively, could achieve steric stabilization of the developed nano-dispersions (Studart et al. 2007; Wiśniewska et al. 2015).

#### Encapsulation efficiency

CS and CS–OA nanoparticles showed mean drug EE of 61.37% ± 0.21 and 60.25% ± 0.15, respectively. EE of 20% was previously reported for CUR-cyclodextrin encapsulated CS nanoconjugates (Popat et al. 2014). Moreover, CUR nanocomplex was prepared at 80% encapsulation efficiency as CUR–CS nanoparticles of the complex (Nguyen et al. 2015). The encapsulation efficiencies of CUR–CS–OA and CUR–CS nanoparticles were similar, implying that CS chains had the predominant influence on the CUR encapsulation. However, the encapsulated CUR in CS–OA nanoparticles was more amorphized as found and discussed later for further nanoparticle testing and characterization (PXRD and SEM analyses), which was explained based on the enhanced CUR-CS interaction mediated by OA (Dhayalan et al. 2024).

#### PXRD and FT-IR

**Fig. 5:**
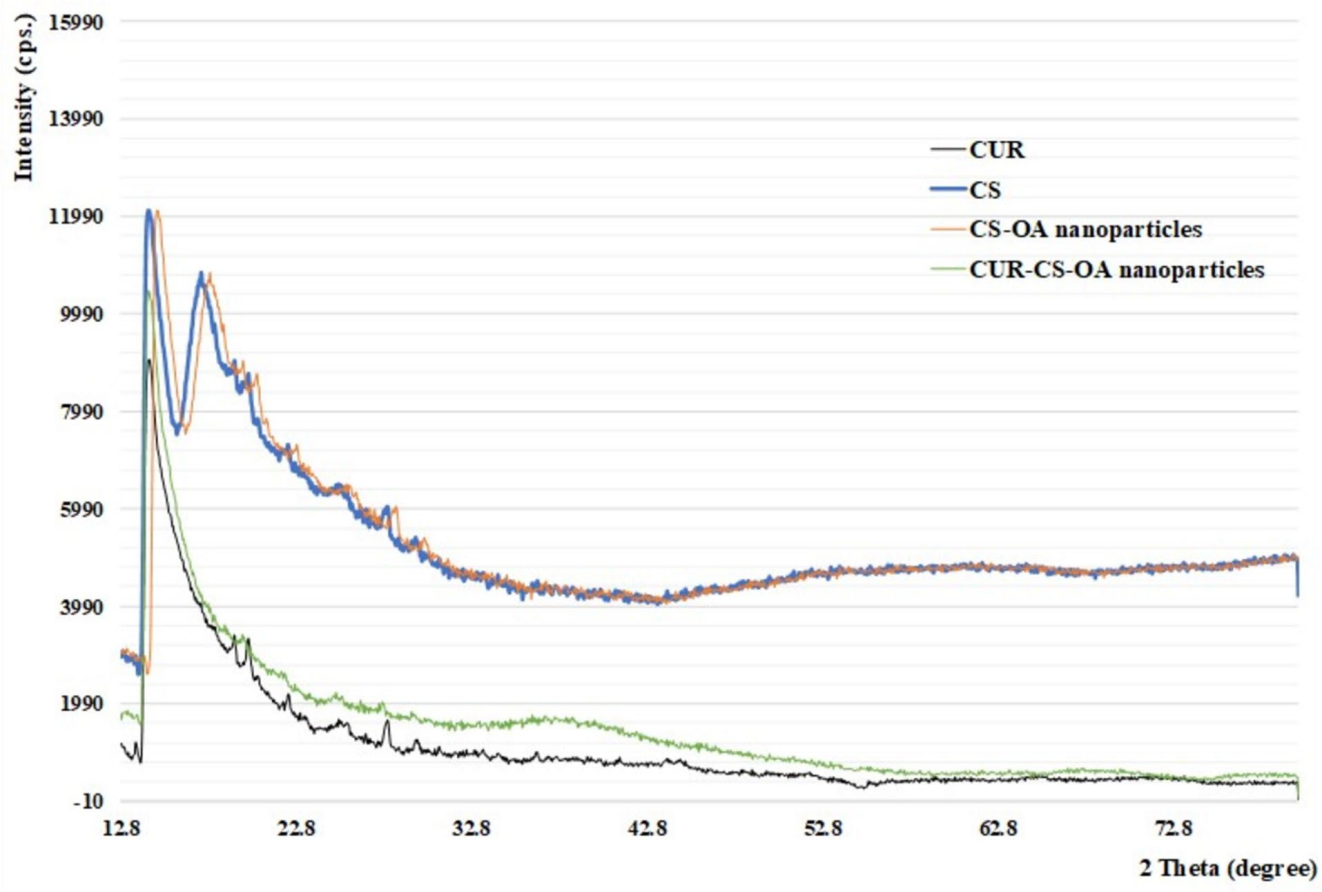
PXRD diffractions of CUR, CS, CS–OA nanoparticles and CUR–CS–OA nanoparticles confirm the amorphization of CUR–CS–OA nanoparticles

The X-ray diffractograms of pure CUR, void CS–OA nanoparticles and CUR–CS–OA nanoparticles are shown in Fig. 5. Numerous diffraction peaks of CUR were observed indicating its crystalline nature. The dried powder of void CS–OA nanoparticle was amorphous where it showed only a few peaks with weak intensities. Almost all CUR peaks vanished in the diffractogram of CUR–CS–OA nanoparticles, which suggested complete amorphization of CUR upon its encapsulation in the nanoparticles. Being both lipophilic, OA might have facilitated the amorphization of CUR by allowing for strong CUR–nanoparticle interactions.

In Fig. 2, the FT-IR spectra of CS–OA and CUR–CS–OA nanoparticles were similar with identical peaks. The broad peak from 4000 to 3000 cm^−1^ in both spectra confirms the hydrogen bond formation between the O–H and N–H groups of CS and the carboxylic groups of OA (Abdullah et al. 2022b). In addition, the peaks from 1846.00 to 1460.00 cm^−1^ in the spectra of CS–OA and CUR–CS–OA nanoparticle groups were correlated to the CS peaks in the same region. The results were supportive of PXRD and zeta potential findings that suggested the surface coating of the CUR by OA and CS though their interaction (Mourdikoudis et al. 2018).

#### SEM and TEM

**Fig. 6:**
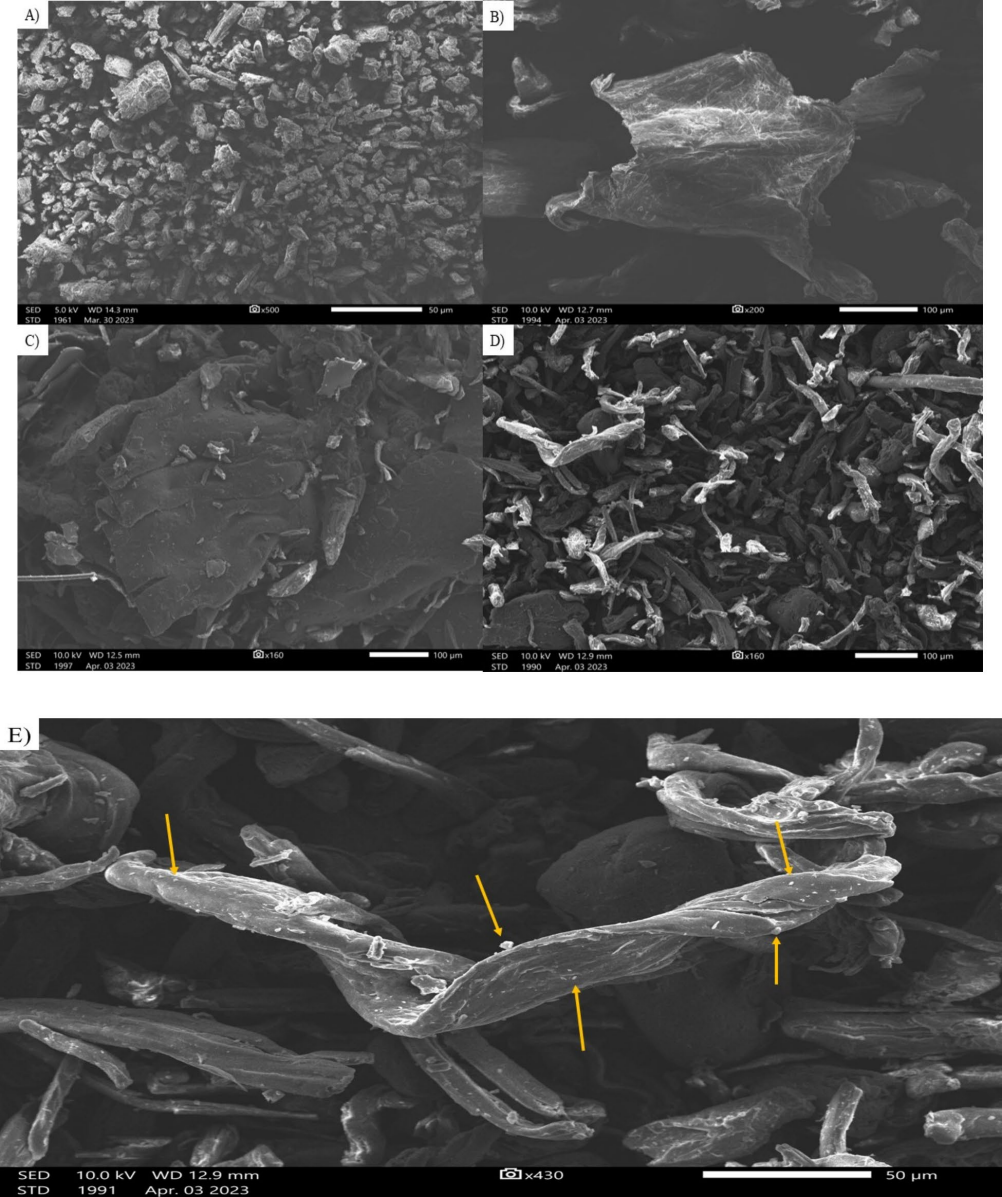
SEM images of CUR (**A**), CS (**B**), CS–OA nanoparticles (**C**), CUR–CS–OA nanoparticles (**D**) with scale bar 50–100 µm, and CUR–CS–OA nanoparticles (**E**) with scale bar 50 µm to confirm the CUR nanoparticles formation on the sheaths of CS–OA

Both dried CUR-CS and CUR–CS–OA nanoparticles displayed irregular CS sheet-like structures, as seen in Fig. 6C, D, respectively. The later product looked to be more discrete, most likely as a result of CS–OA interactions. CUR powders scattered among CS sheets were visible at 160 X magnification when OA was absent (Fig. 6D). Such particles could not be identified for CS–OA nanoparticles at the same magnification (Fig. 6C); however, a small number of CUR particles appeared with a higher magnification of 430 X (Fig. 6E). The findings indicated that there was less phase separation between CUR and CS-OA, most likely because OA increased the ability of CS to solubilize CUR.

**Fig. 7:**
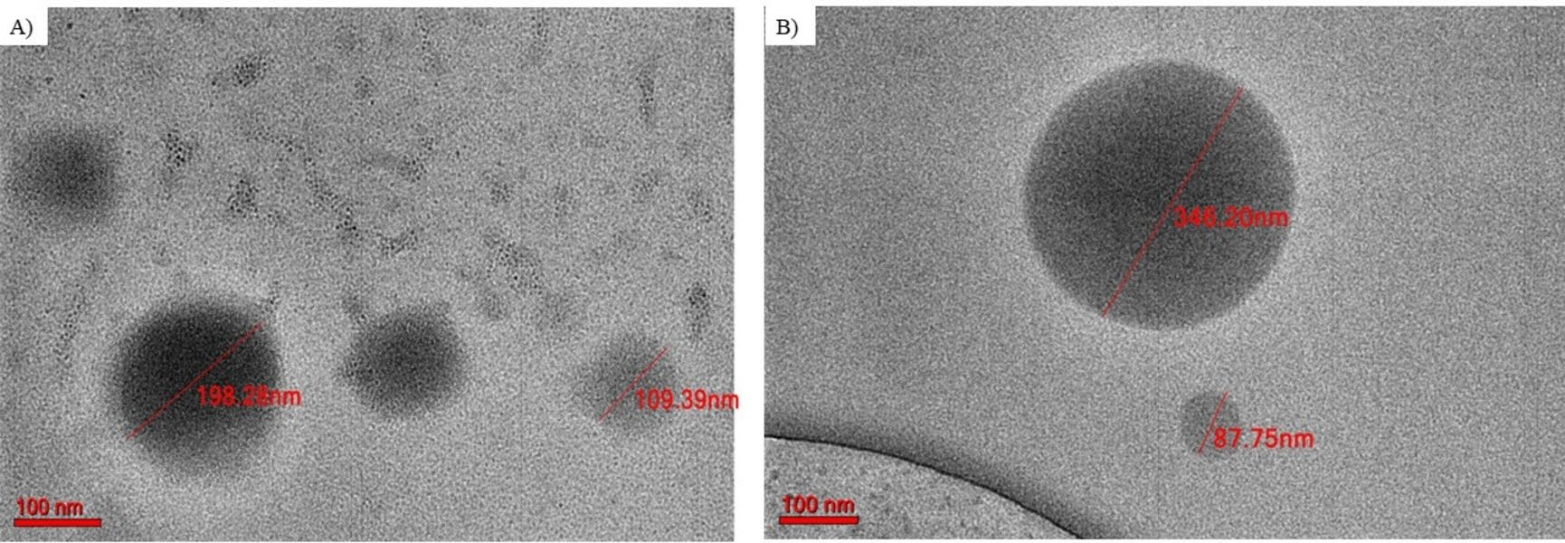
TEM images of **A** CUR–CS–OA nanoparticles and **B** CS–OA nanoparticles with a scale bar of 100 nm. The CUR–CS–OA nanoparticles' size is lower than the CUR–CS nanoparticles

TEM images of the CUR–CS–OA nanoparticles and blank CS–OA nanoparticles (Fig. 7A, B) showed spherical nanoparticles with sizes in the range 109.00–198.00 nm and 87.75–245.25 nm, respectively. The results came in consistent with the particle size analysis results. Based on the FT-IR, PXRD, and TEM images, the OA influence was based on stabilizing the interactions between the hydrophobic CUR and the cationic CS polymeric chains, which allows for the development of neat and spherical nanoparticles (Dhayalan et al. 2024).

### Biocompatibility and cytotoxicity

CUR significantly reduced the cell viability of prostate cancer cell lines (DU145 and PC-3 cell lines) by inducing cell death through different mechanisms. It was suggested that cell death occurred by producing cytoplasmic vacuoles as a non-phagocytic behavior (Lee et al. 2015). Another study suggested that CUR is a chemo-preventive agent for early-stage prostate cancer (Teiten et al. 2011).

**Fig. 8:**
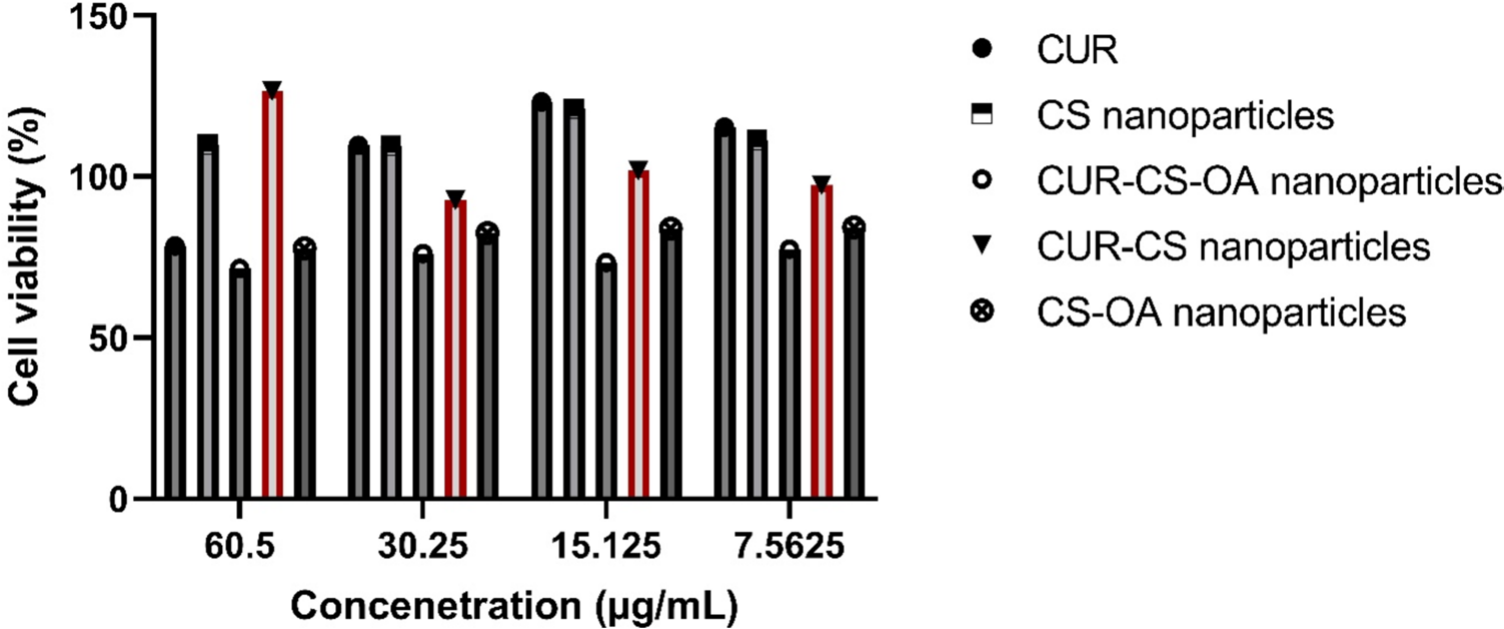
Cell viability of human dermal fibroblasts (HDFa-cells) exposed to different concentrations of CUR solution, CUR–CS nanoparticles, CS nanoparticles, CUR–CS–OA nanoparticles and CS–OA nanoparticles. Data presented are mean ± SD of triplicate values. This figure indicates the biocompatibility results of all groups

Human dermal fibroblasts (HDFa-cells) were used to test the cytotoxicity of CUR-CS and CUR-CS-OA nanoparticles concerning CUR solution and the corresponding blank nanoparticles to ensure their safety (Kisiel and Klar 2019). The cell viability of HDFa- cells was not significantly less than 80% at any of the concentrations evaluated, as shown by the results in Fig. 8. This suggests that the polymers employed to make the nanoparticles were safe. The negatively charged phosphate surface of the cells may allow for improved cellular adhesion, penetration, and absorption due to the nanoparticles' positive surface charges. However, due to safety concerns and unregulated cellular uptake, the nanoparticles' surface positive charges are favorable up to a certain point. Thus, the examined nanoparticles' low zeta potential may account for their safety (Mourdikoudis et al. 2018). TEM and particle size analysis results of the CUR-CS-OA and CUR-CS nanoparticles show lower sizes of CUR-CS-OA than CUR-CS nanoparticles, which may encourage the cellular internalization and uptake of CUR-CS-OA nanoparticles (Bravo-Osuna et al. 2007).

**Fig. 9:**
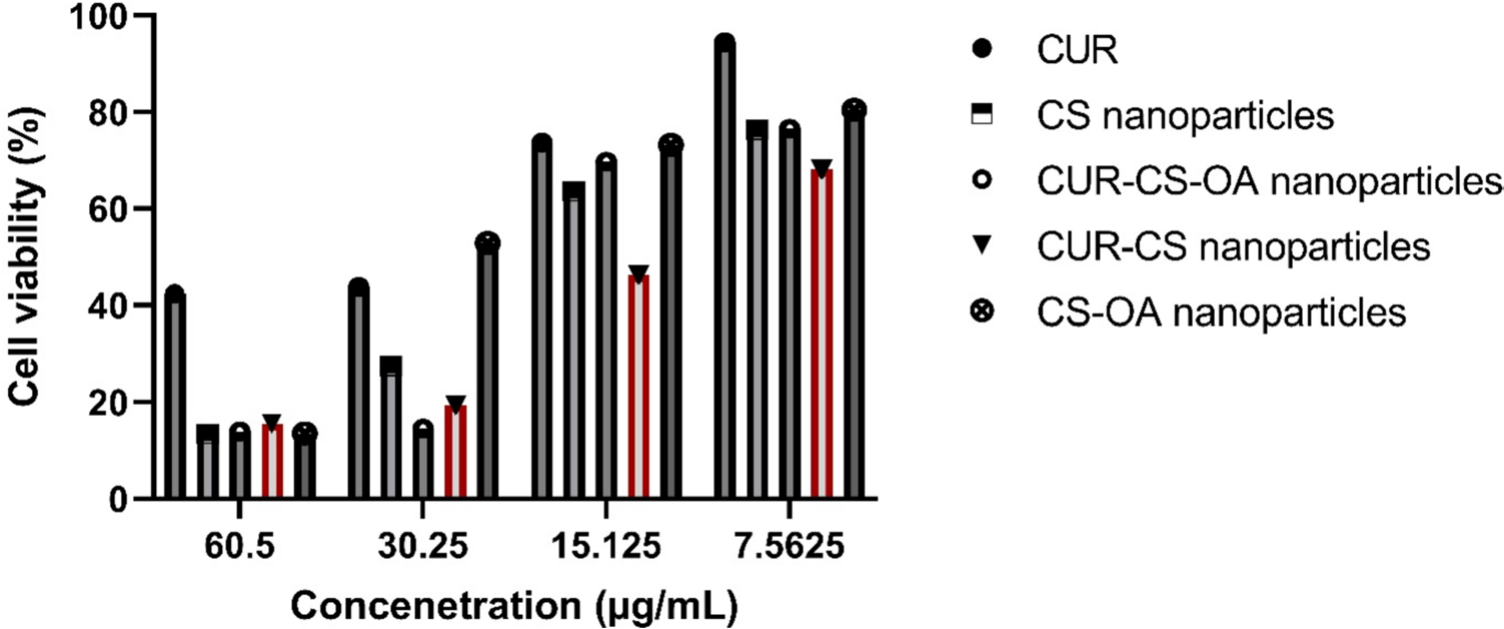
Cell viability of prostate cancer cells (DU145) exposed to different concentrations of CUR solution, CUR–CS nanoparticles, CS nanoparticles, CUR–CS–OA nanoparticles and CS–OA nanoparticles. Data presented are mean ± SD of triplicate values. This figure indicates superior CUR–CS–OA nanoparticles' anticancer abilities in comparison to the other groups over the different concentrations

**Fig. 10:**
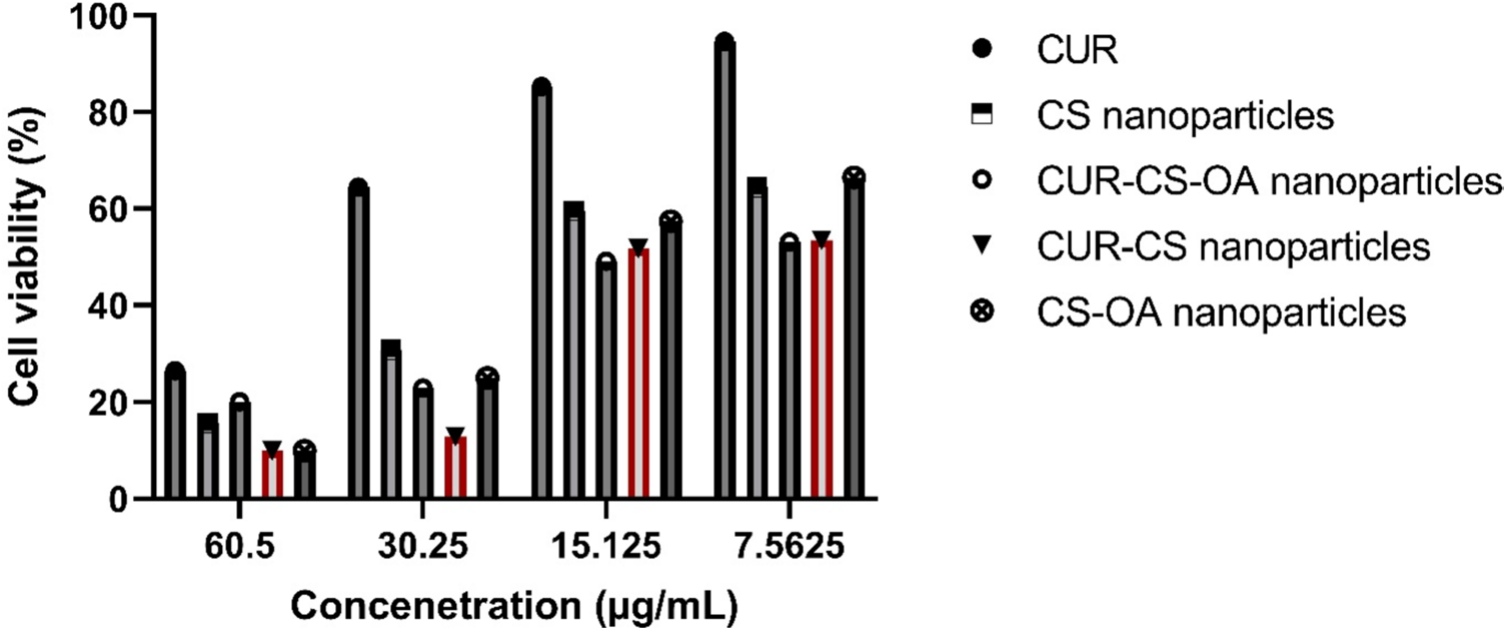
Cell viability of prostate cancer cells (PC3) exposed to different concentrations of CUR solution, CUR–CS nanoparticles, CS nanoparticles, CUR–CS–OA nanoparticles and CS–OA nanoparticles. Data presented are mean ± SD of triplicate values. This figure indicates superior CUR–CS–OA nanoparticles' anticancer abilities in comparison to the other groups over the different concentrations

Regarding the anti-cancer activity, the blank CS and CS-OA nanoparticles showed antiproliferative activity against both prostate cancer cell lines (DU145 and PC3), which was concentration-dependent and greater than that of the CUR solution, particularly at higher concentrations (Figs. 9 and 10). Despite that CS is considered a biocompatible and biologically safe polymer, its nanoparticles without any cytotoxic agent incorporated (blank) were demonstrated by several reports to have cytotoxicity, which was dependent on the cell line type, particle size, and concentration and particularly favors cancerous cells as compared to non-cancerous cells. CS cytotoxicity is mediated by adhesion to the cell membrane facilitated by the electrostatic interaction between the polymer's positive charge and negatively charged cellular components, followed by cellular internalization. The antitumor mechanism of CS nanoparticles was related to its membrane-disrupting and apoptosis-inducing activities. Examples of the profound cytotoxicity of blank CS nanoparticles are the antiproliferative activities of void CS nanoparticles shown against cancer cell lines of breast cancer, T lymphocyte acute leukemia, sarcoma, hepatocellular carcinoma and gastric carcinoma. The cytotoxicity of the blank CS nanoparticle against prostate cancer cell lines is in support of the findings in these previous studies (Qi et al. 2005; Aibani et al. 2021; Jiang et al. 2018; Thandapani et al. 2017).

A careful examination of Figs. 9 and 10 can show that, in general, CUR loading enhanced the cytotoxicity of the blank nanoparticles at the different tested concentrations. In support of this, for DU145 prostate cancer cells, mean IC_50_ values of 20.21, 19.21, 15.41, 17.63, 14.33 and 25.43 µg/mL were calculated for CUR, CS nanoparticles, CUR-CS nanoparticles, CUR-CS-OA nanoparticles and CS-OA nanoparticles, respectively. In addition, for PC3 prostate cancer cells, the respective IC50 values were 40.03, 17.32, 15.11, 20.33, 13.07 and 18.41 µg/mL for CUR, CS nanoparticles, CUR-CS nanoparticles, CUR-CS-OA nanoparticles, and CS-OA nanoparticles, respectively. These comparisons also point out that among other tested preparations, CUR-CS-OA nanoparticles having the lowest IC_50_ value were the most potent against prostate cancer cells. The prostate cancer cell membrane is overexpressed with cholesterol, which could lead to a higher interaction between the hydrophobic CS-OA nanoparticles and the prostate cancerous cell membrane (Freeman and Solomon 2004).

## Methodology

### Materials

The following materials were purchased from Sigma Aldrich (USA). CUR powder, bovine albumin fraction V (98–99%), citric acid, dialysis tubing made of cellulose membrane with 12,000 to 14,000 cut-off value and an average flat width of 25 mm, dimethyl sulfoxide (DMSO), high molecular weight CS, MTT assay kit, octanoic acid (OA), polyvinyl alcohol (PVA), and sodium triphosphate pentabasic (TPP).

### CS-OA salt formation

#### Optimum OA:CS ratio

High-molecular-weight CS was dissolved at a concentration of 10 mg/mL in 2.5% citric acid. A clear K octanoate solution was obtained by combining OA (3 g) and an equimolar volume of KOH (0.3 M, 70 mL) in a beaker at room temperature with stirring. In Eppendorf plastic tubes (50 mL), CS solutions (30 mL) were mixed with various volumes (between 4 and 20 mL) of K-octanoate solution to create mixtures with various CS:OA volume ratios as shown in Table 2. With distilled water, fixed final quantities of 50 mL were attained. The mixtures were shaken for 30 min at 9150 rpm in a shaker incubator at 25 °C, homogenized for 30 s, and then centrifuged for 10 min to give distinctive coprecipitates and clear supernatants that were separated by decantation. The mixtures were shaken for 30 min in a shaker incubator at 9150 rpm and a temperature of 25 °C, homogenized for 30 s and centrifuged for 10 min at 4000 rpm.

**Table 2:** Composition of aqueous mixtures of chitosan citrate and K-octanoate

Mixture #	CS citrate solution (mL)	K octanoate solution (mL)	Distilled water volume (mL)
2	30	4	16
3	30	8	12
4	30	11	9
5	30	12	8
6	30	16	4
7	30	18	2
8	30	20	0

#### Preparation and characterization of CS–OA salt

CS–OA salt was prepared by the coprecipitation method according to the procedure described above using the optimum weight ratio of salification corresponding to mixture 6 in Table 2. The isolated coprecipitate was double-washed with distilled water and then dried at room temperature in a glass Petri Dish under vacuum. CS–OA salt formation was assessed by observing changes in infra-red absorption of the coprecipitates relative to those of CS, OA, and their physical mixture. For this purpose, Fourier-transform infrared (FT-IR) spectra were obtained using an FT-IR spectrometer (Agilent Technologies Carry 630 FT-IR, USA). A suitable amount of each sample was constantly applied on the crystal plate surface of the device to cover the entire surface of the prism. The samples were scanned from 500 to 4000 cm^−1^.

### Nanoparticles preparation

#### CUR-CS nanoparticles

The ionotropic technique was used to prepare the nanoparticles. First, CS (100 mg) was dissolved in a 4% citric acid solution (35 mL). Under stirring, 10 mL of PVA solution (2%) was added with the produced CS solution. DMSO (2 mL) was used to dissolve CUR (20 mg). Of this solution, 1 mL was added dropwise to the CS-PVA mixture under vigorous stirring. Afterward, tripolyphosphate (TPP) aqueous solution (10 mg/5 mL) was added dropwise under stirring. After further stirring for 30 min, the dispersion was loaded into a membrane dialysis bag with tightly closed ends that dialyzed for 1 h to remove DMSO and free unloaded CUR (Studart et al. 2007).

#### CUR-CS-OA nanoparticles

CS (100 mg) and bovine albumin serum (BSA) (25 mg) served as nanoparticle stabilizers were dissolved in 2% citric acid aqueous solution (50 mL). CUR (10 mg) and OA (0.25 mL) were dissolved in DMSO (0.75 mL). This solution was added dropwise to the CS-BSA solution under stirring. The resulting dispersion was dialyzed as described above for CS nanoparticles (Studart et al. 2007).

### Nanoparticle characterizations

#### Size analysis and zeta potential

The dynamic light scattering method (DLS, Malvern Nano-ZEN 3600, UK) was used to measure the particle size and zeta potential of the nano-dispersions. Before measurement, the dispersions were diluted (1:1) with phosphate buffer (pH 7.4), and the measurements were conducted at 25.00 ± 0.05 °C. All measurements were performed in triplicate (Abdullah et al. 2022a).

#### Encapsulation efficiency

In volumetric flasks, accurate volumes (1 ml) of the nano-preparations were added to 25 ml of absolute ethanol and mixed to produce transparent solutions. At 421 nm, the UV absorbance of the solutions was measured. Using an appropriately constructed calibration curve equation, CUR concentrations and quantities were determined from the absorbance. The following equation was used to compute the percentages of CUR encapsulated in the nanoparticles:

$$\text{Encapsulation efficiency }\left(\text{\%}\right)=\frac{Wt}{Wi}\times 100$$

where Wt is the CUR amount encapsulated in the nanoparticles and Wi is the initial quantity of CUR used for nanoparticle preparation. The experiments were performed in triplicate (Md et al. 2022).

#### Powder X-ray diffraction (PXRD) and fourier-transform infrared spectroscopy (FT-IR)

The study employed PXRD equipment (Rigaku, Japan) to examine CUR, blank CS–OA nanoparticles, and CUR–CS–OA nanoparticles. Using a nickel filter and Cu-Kb reduction, X-rays were produced at 40 kV and 100 mA. The scan range (2*θ*) was 5–70 degrees at a speed of 10 degrees per minute. Fourier-transform infrared (FT-IR) spectra of the nanoparticles were obtained using an FT-IR spectrometer (Agilent Technologies Carry 630 FT-IR, USA).

#### Scanning electron microscopy (SEM) and Transmission electron microscopy examinations (TEM)

The examined nanoparticle dispersions were dried using heat fixation on a glass slide before SEM platinum coating and imaging. The morphology and particle size distribution of the dried powders of blank CS–OA nanoparticles and CUR–CS–OA nanoparticles, as well as CUR and CS powders, were assessed using SEM (Jeol, Japan) at 20 kV voltage. The shape and size of the nanoparticles were examined using TEM (JEM-F200, Jeol, Japan). Once the sample dispersions had been diluted using an ultra-pure water (1:50) ratio, one drop of the diluted dispersion was placed onto a copper grid to achieve negative staining (phosphotungstic acid, 2%).

### In vitro biocompatibility and cytotoxicity

#### Cell culture

One vial each of PC3 (ATCC® CRL-1435™), DU 145 (ATCC® HTB-81™) as human prostate cancer cell lines, and Fibroblast (HDFa) cells were obtained from the Hamdi Mango Center for Scientific Research, the University of Jordan, Amman, Jordan. All cell lines were grown in recommended media and specified additives according to source, the American Type Culture Collection (ATCC™). The cells were cultured in Dulbecco’s Modified Eagle Media (DMEM) (Euro-Clone™, Italy) supplemented with 10% fetal bovine serum (FBS) (Biowest, USA), penicillin (100 IU /mL) and streptomycin (100 µg/ mL) (Euro-Clone™, Italy) under strict sterile conditions at 37 °C in a humidified environment containing 5% carbon dioxide (CO_2_) incubator. The cells were harvested when the flask showed 70–90% confluency. The attached cells were washed with phosphate-buffered saline and harvested by Trypsin (0.25%)/EDTA (0.04%) at 37 °C in a CO_2_ incubator for 10 min. After the detachment of cells, 10 mL quenching medium was added to the flask, and the harvested cells were added to a 15 mL centrifuge tube and centrifuged at 1100 rpm for 7 min. After the formation of the cell pellets, the cells were dispersed in a fresh, warm tissue culture medium and counted using Trypan blue and a hemocytometer.

#### MTT assay

The preparations tested were CUR–CS nanoparticles and CUR-CS-OA nanoparticles, in comparison with the corresponding void nanoparticles and CUR hydroalcoholic solutions. Each preparation was tested at equivalent CUR concentrations in the range of 7.57–60.50 µg/mL. CUR nanoparticles were diluted with the tissue culture medium to provide the tested concentrations. The vehicle of CUR solution and void nanoparticles were also diluted similarly to provide controls (Nguyen et al. 2015).

The DU145 and PC3 cancer cell lines, as well as the HDFa fibroblast cells under examination, were plated in 96-well plates at a density of 5 × 10^3^ cells per well in 100 μL of DMEM with 10% FBS and allowed to grow for 24 h. Following that, cells were exposed to a range of doses of free CUR, CUR-encapsulated nanoparticles, or CUR-free for 72 h. The MTT assay was used to assess the cell viability after 72 h of incubation. The supernatant on top of the cells was decanted, and 100 µL of fresh media was added over the cells, and then 10 µL of the MTT dye was added to each well. The dye was removed by a thin needle syringe, and then 100 µL of DMSO as a solubilizing agent was added to each well. The plates were shaken for 5 min at 100 rpm and then the absorbance of the dye was measured using a microplate UV-reader (BioTek instrument, USA) at a wavelength of 570 nm. The viability of cells was calculated for the hydroalcoholic solution of CUR after correction to the blank of ethanol by dividing the absorbance measurement of cells in CUR solution by those in ethanol, expressed as the average. For the nanoparticles, the average absorbance is correlated to the cell viability as high measurements of dye absorbance mean high cell viability and less cytotoxicity.

## Conclusions

OA can be used as CS salt former for the sake of converting the hydrophilic polymer to a hydrophobic one. The salt has the ability to amorphize CUR and produce nanoparticles with high encapsulation. The nanoparticles' amorphization of CUR suggests that OA improved the interactions between CS and the hydrophobic CUR. Additionally, it is feasible to manufacture nanoparticles without the use of chemical or inorganic cross-linkers, which is beneficial when taking into account the potential toxicity of such cross-linkers. Both CS–OA and CS void nanoparticles are cytotoxic to prostate malignant cells, suggesting polymeric cellular internalization. The cytotoxicity of these nanoparticles was further augmented by loaded CUR, particularly into CS-OA nanoparticles. The new tested and characterized nanoparticle platform can be further tested in vivo for stability and biodistribution. It can be also evaluated for other cytotoxic drugs and in other cancerous cells.

## Data Availability

Data are available on request.
